# Improving Adherence to Treatment in Patients With Diabetes: Practical Strategies

**DOI:** 10.1002/edm2.512

**Published:** 2024-07-12

**Authors:** Donya Sadeghi

**Affiliations:** ^1^ Faculty of Nursing and Midwifery Tehran University of Medical Sciences Tehran Iran

Dear Editor,

Diabetes is one of the most common metabolic diseases worldwide [1, 2]. In addition to complications such as retinopathy, nephropathy, neuropathy, repeated infections or even limb amputation, lack of continuous blood sugar control can cause economic problems and disruption in the progress of prescribed treatments for patients [3]. According to the World Health Organization, adherence to treatment includes behaviours such as taking medications, following a diet, and living a healthy lifestyle that are consistent with the recommendations of health care providers. Despite the great advances of medical science in the treatment of diabetes, the poor adherence of patients to the treatment and prescribed drug regimen is still considered as one of the important challenges related to this disease. Adherence to treatment is simultaneously influenced by several factors such as social and economic factors, healthcare team/system‐related factors, condition‐related factors, treatment‐related factors and patient‐related factors [4]. Therefore, to improve treatment adherence, issues related to each factor must be addressed. The aim of the present study was to provide practical strategies to improving adherence to treatment in patients with diabetes.

Social problems of patients with diabetes include feelings of fear, embarrassment, blame, guilt, anxiety and decreased self‐confidence, which may lead to a feeling of social stigma in the patient. For this reason, these patients may resort to strategies such as hiding the disease, not performing therapeutic activities in public, avoiding social relations and finally not adherence to treatment of their disease [5, 6]. Meanwhile, family support, as one of the most important sources of social support, plays a significant role in increasing self‐confidence and adaptation to the disease in people with diabetes. The coordination and cooperation of family members, especially in relation to understanding the physical and mental conditions of the patient, following the diet and remembering to take the appropriate and timely dose of medicine, helps a lot to adherence to treatment in the patient with diabetes [7]. In this regard, one of the most important ways to increase compliance with treatment is to increase the level of awareness of the patient, family and society about the nature, control and transmission of this disease. Educational interventions to improve the level of health literacy, based on educational strategies, including lectures, audio and video media, interviews, the use of mobile phone software programs, social networks, as well as low‐cost and accessible education such as the use of SMS By raising the level of understanding and knowledge, it can lead to continuous adherence to treatment and increase the patient's self‐confidence in enjoying their normal life [2, 8].

It is not a secret that the diagnosis and control of diabetes and its acute and chronic complications require spending significant costs on behalf of patients (visits, tests and drugs) as well as the health care system of society. On the other hand, a decrease in income due to a drop in productivity or the patient's inability to perform the tasks assigned to her can also cause his/her family's economy to face many problems. This causes the patient's inability to cover the costs of the disease and ultimately nonadherence to treatment in the long term [3]. In this regard, the expansion of nongovernmental organisations (NGOs) by providing services to people with diabetes can be very useful in compensating for the damages caused by this disease. Another effective strategy to minimise economic losses in patients with diabetes is to cover all processes related to the diagnosis and treatment of this disease by governments. Due to the limited resources to meet healthcare needs in most countries, careful planning is necessary to use these resources. Cost control and targeting should be at the top of governments' plans in their behaviour towards healthcare systems and significant efforts should be spent on analysing the costs and damages caused by the disease and its complications in order to facilitate the planning of health and treatment services for patients with diabetes.

Nowadays, environmental conditions and unhealthy lifestyle have gradually exposed people to chronic diseases. Healthcare centres are also not easily accessible for patients with diabetes in many low‐and‐middle‐income countries [4]. This disease requires long‐term medical care to prevent acute complications and reduce the risk of long‐term complications. This is while many patients with diabetes living in remote areas do not have access to the most essential and even the most basic facilities needed to monitor and treat their disease. This causes the regular adherence to treatment in these patients to be disturbed unintentionally and the ground for the occurrence or exacerbation of the complications of their disease is provided [1, 9]. The most practical strategy is that the targeting of health systems regarding optimal diagnosis and treatment programs for patients with diabetes should be based on universal coverage in order to prevent complications and disabilities through adherence to treatment and continuous care.

Patients with diabetes always need consultation and interaction with their treatment team to get the necessary recommendations for disease management and the motivation to adherence to their treatment plan. Therefore, the strong performance of the treatment team in terms of responding appropriately to the needs of patients, providing complete information and away from contradictions based on the defined protocols, as well as trying to accompany patients in care plans, can gain their trust and satisfaction and provide them with the opportunity to refer again and adherence to treatment [6]. Another effective strategy to improving adherence to treatment is to adopt a collaborative approach in the behaviour of health personnel with clients. In other words, any decision regarding how to implement the treatment protocol should be made with the patient, not instead of the patient. Creating a broad behavioural change and adherence to a new care and treatment regimen to control diabetes requires a targeted and efficient effort from the patient and the treatment team [2]. Forcing the patient to make these changes jeopardises the healing process and, subsequently, compliance with the adherence to treatment. Therefore, the treatment team can gradually change the patients' lifestyle and increase their motivation in self‐care and continuous adherence to treatment, according to the health beliefs and the understood concepts of the patients regarding the disease.

The ultimate goal of diabetes treatment is to control blood sugar at a normal level, and a major part of the treatment process is done by the patient rather than the treatment team. Therefore, the beliefs related to the controllability of the disease and the complications caused by it and self‐care of patients is significantly effective in the medication adherence of people with diabetes. The patient's positive attitude towards the effect of the drugs used in reducing the complications of the disease encourages his/her to continue the treatment process and increase her ability to take care of herself. The promotion of self‐care activities, which includes various dimensions such as physiological, emotional and spiritual, can play an effective role in disease control. This indicates that diabetes is closely related to psychological phenomena, and in the meantime, its two‐way relationship with stress is very important [10]. Disappointment in the effectiveness of treatment, job burnout, dependence on others, old age, depression and imposing an additional burden due to treatment often provide difficult and stressful conditions for patients with diabetes, which prevents continuous adherence to treatment. In this regard, self‐management is an individual solution that can be useful in minimising the emotional load resulting from stress in patients and prevent negative behaviours in them [7]. Therefore, self‐management is an effective tool that includes physical activity, healthy eating, adherence to treatment and medical orders, regular blood glucose monitoring and personal health‐related problem solving, and its proper training to patients with diabetes can play an effective role in their healthy lifestyle.

A review of numerous studies shows that providing practical strategies to improve adherence to treatment in patients with diabetes requires a perspective in which the patient, family, treatment team and society are considered as main elements and interrelated circles. It seems that various interventions to strengthen each of these elements alone, have not been effective in improving the patient's condition in a long‐term and complete way in following the prescribed treatment process. Perhaps this is why adherence to treatment still remains a challenge for medical professionals and healthcare providers in many countries.

## Data Availability

Data is available on request from the authors. If anyone wants to request the data from this study, they should contact Donya Sadeghi (d-sadeghi@student.tums.ac.ir).
